# Building Chemistry from “Lego” Blocks: Metal–Organic Frameworks after the 2025 Nobel Prize

**DOI:** 10.1021/acscentsci.6c00758

**Published:** 2026-07-01

**Authors:** Grace C. Thaggard, Danielle N. Smith, Sumanta Basak, Buddhima K. P. Maldeni Kankanamalage, Amanda J. Morris, Natalia B. Shustova

**Affiliations:** † Department of Chemistry and Biochemistry, 2629 University of South Carolina, Columbia, South Carolina 29208, United States; ‡ Department of Chemistry, 1757 Virginia Polytechnic Institute and State University, Blacksburg, Virginia 24061, United States

## Abstract

Leading up to the 2025 Nobel Prize in Chemistry, the field of metal–organic frameworks (MOFs) shifted the material design paradigm by allowing for discrete molecular building blocks to be extended into predesigned porous functional materials. Outside of developing an expansive library of crystal structures and fundamentals of structure–property relationships, application-driven MOF research has led to commercialization of MOF-based materials for direct air capture of CO_2_, atmospheric water harvesting, and protection of first responders. Today, the MOF field addresses urgent global challenges related to clean water scarcity, sustainable energy, disease treatment, and advanced technology. This Outlook highlights some of the future MOF research avenues that could define the post-Nobel Prize era of framework chemistry, beginning with the implementation of controlled chemical heterogeneity in well-defined structures to unlock heterogeneous tandem catalysts or materials for gradient drug release. Upcoming studies of adaptive and/or magnetic frameworks will bring MOFs to the forefront of quantum materials research. Finally, this Outlook surveys some of the key challenges to address to expand MOF commercialization. The early years of reticular chemistry have provided the conceptual “blocks” to build a new generation of frameworks that will reshape the materials landscape, from innovative synthetic strategies to quantum computing and beyond.

## Graphical abstract

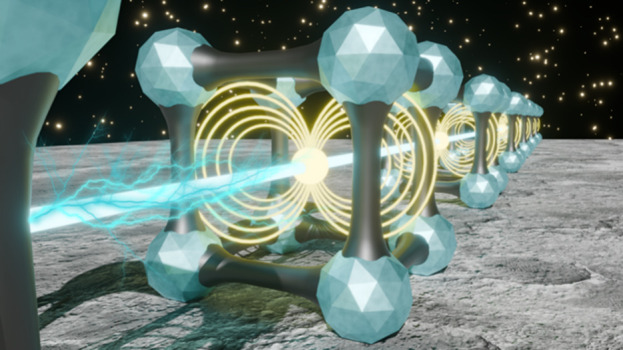

## Introduction

In the years leading up to its recognition with the 2025 Nobel Prize in Chemistry, the field of metal–organic frameworks (MOFs) has applied the guiding principles of reticular chemistry to continually evolve from fundamental studies of framework synthesis to the targeted design of advanced functional materials.^1^ Concepts that enable the predictable connection of discrete molecular building blocks into extended structures with predesigned topologies, pore architectures, and functionalities have fundamentally transformed the paradigm of material design.^2–4^ In addition to generating an expansive library of crystal structures, the MOF field has established an innovative conceptual framework merging molecular-level properties with macroscopic material performance. This “from molecules to functional materials” perspective has allowed for the rational design of high-performance MOFs,^5–11^ some of which have already been commercialized for direct air capture,^12,13^ hazardous gas storage,^14,15^ atmospheric water harvesting,^16,17^ and dehumidification applications.^18,19^ As of 2025, more than 45 start-up companies and over 4,000 patent applications are leveraging MOF chemistry to address pressing global challenges, including water scarcity, rising greenhouse gas emissions, growing demand for renewable energy, and disease treatment.^5,20–22^ Additionally, MOF research is actively working toward solutions for some of the important issues in the petrochemical industry, such as adsorption-based separation and purification of hydrocarbons, directly impacting the global economy.^23,24^ With more than 100,000 reported crystal structures constructed from over 10,000 organic linkers and more than 66 metals,^5,25^ the MOF field is poised for continued rapid expansion in both fundamental understanding and technological applications. Building on these advances, this Outlook highlights several emerging frontiers in MOF research, including molecular-level perspectives on porous frameworks, the development of adaptive and quantum materials, and the continual commercialization of MOF-based materials.

## Framework Heterogeneity: From Uniform Hosts to Programmable Chemical Microenvironments

One of the concepts likely to define the next steps in MOF chemistry is the deliberate introduction of chemical/compositional heterogeneity (e.g., variable linker functionalities or metal node compositions) within well-defined frameworks (Figure 1). Beyond structural exploration, early MOF research strategies primarily focused on the ability to predictably synthesize crystalline materials with a “homogeneous” distribution of chemically active sites, i.e., structures in which all metal sites possess identical coordination environments and chemical characteristics. More recently, however, approaches inspired by biological systems, such as enzymes, have demonstrated clear advantages of “controlled heterogeneity”, i.e., rationally imposed chemical heterogeneity within MOF structures.^26–28^

**Figure 1:**
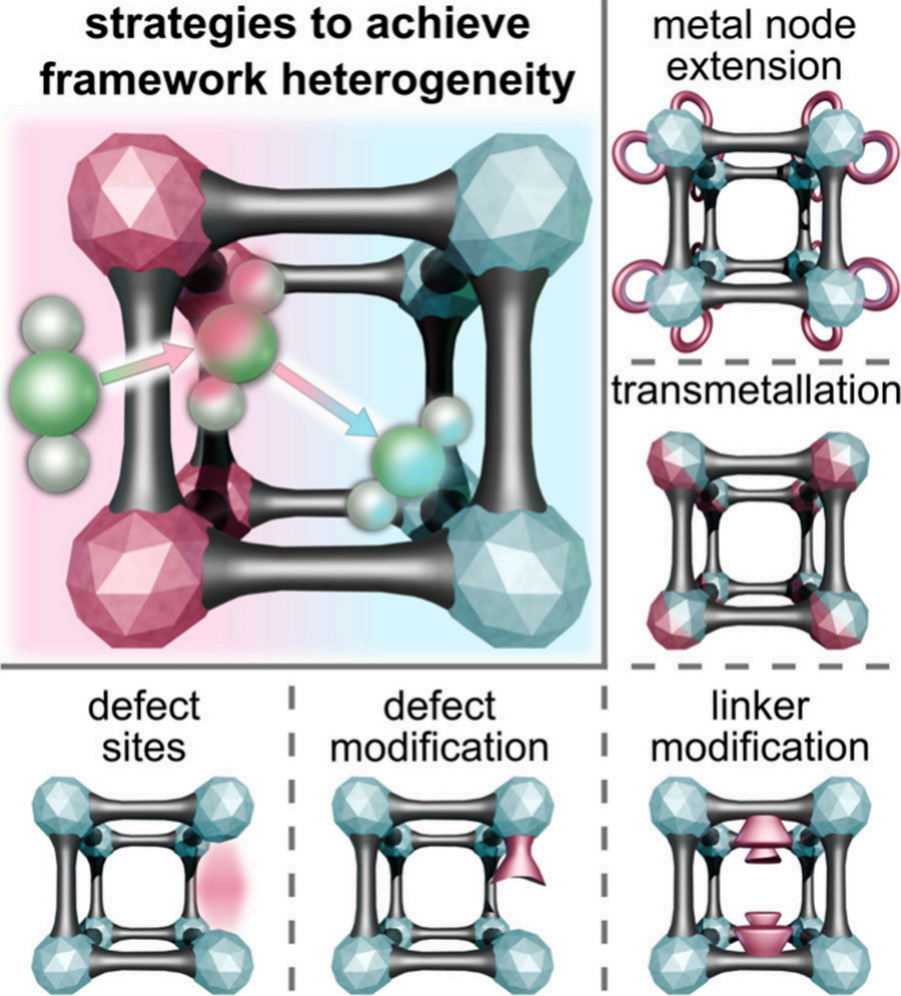
Schematic representation of a chemically heterogeneous framework. The presence of different metal node compositions (pink and blue polyhedra) promotes variable substrate diffusion pathways, as well as binding and release of guest molecules through MOF channels. Possible synthetic strategies to achieve a chemically heterogeneous framework are highlighted, including metal node extension, transmetalation, presence of defect sites, and defect/linker modification.

In principle, the defined topology of a given MOF could be used to impose cooperation between chemically distinct linkers, metal nodes, or defect sites through their spatial organization, which could be advantageous for multistep catalytic activity, selective separations, or controlled drug delivery.^29–31^ Thus, one of the future directions in MOF chemistry research will be to integrate chemical heterogeneity not only as a parameter for MOF synthesis but also as a key tool for controlling and programming material functions.

Thus, one of the future directions in MOF chemistry research will be to integrate chemical heterogeneity not only as a parameter for MOF synthesis but also as a key tool for controlling and programming material functions.

There are many diverse functional environments within a chemically “heterogeneous” framework, e.g., different types of alternating metals integrated at the same crystallographic site, structurally similar linkers but with distinct attached functionalities, defect types and numbers,^32–34^ differing pore polarities, and integrated dynamic response elements, all physically distributed throughout the framework.^35–37^ For example, one of the best-known frameworks, MOF-5, can accommodate 36 different linkers modified with 27 different functional groups within a single structure.^36^ In such cases, the overall connectivity and topology of the framework are well-defined and conform to the well-studied MOF-5 structure. However, the chemical environment (i.e., hydrophilic/hydrophobic pores, presence of binding groups, electrostatic interactions, etc.) that a guest molecule would experience upon diffusion through the heterogeneous MOF-5 analog is highly diverse.^36^ The described diversity would increase exponentially upon subsequent postsynthetic modification, such as transmetalation or linker exchange (Figure 1).^29,38^ Such compositional diversity will allow MOFs to do more than just passively adsorb guest molecules; they could also act as catalysts with controlled reactivity based on guest-molecule diffusion pathways regulated by chemical heterogeneity.

A central future direction lies in programming cooperative interactions between neighboring sites by precisely controlling relevant interlinker distances and angles. In most existing multivariable MOFs, functionalities are statistically (“randomly”) distributed throughout the bulk material, resulting in largely independent or noncooperative behavior of the organic linkers. Thereby, to address such uncontrolled distribution, next-generation heterogeneous frameworks will likely be more strategically designed so that one site modulates the behavior of another. For instance, some potential strategies to achieve controlled chemical heterogeneity in MOFs could involve pairing redox-active nodes with linkers that facilitate electron/ion transport. Alternatively, a Lewis acidic site could be positioned adjacent to a Bronsted base to enable tandem catalytic steps within a confined pore environment.^39,40^ Such spatial organization of heterogeneous sites is a feasible pathway for catalyzing multistep transformations or achieving biomolecular recognition. However, access to this level of control will require developing new synthetic approaches that create a preference for targeted spatial arrangements of variable organic linkers or for selective transmetalation of specific metal nodes (Figure 1). As more synthetic methods for accessing chemically heterogeneous (multivariate) MOFs are developed, characterization of the resulting structures through, for instance, powder or single-crystal X-ray diffraction, continuous rotation electron diffraction, or scanning probe microspectroscopy, will also be vital moving forward.^41,42^

Framework “heterogeneity” also offers a powerful strategy for addressing mass transport limitations in porous materials, which often dictate MOF performance across many applications. In catalysis, high intrinsic chemical activity can be overshadowed by slow substrate diffusion through MOF pores or by inefficient charge transfer/separation (i.e., rapid electron–hole recombination rates and/or low charge-carrier mobility).^26^ By integrating hierarchical porosity (i.e., through partial or selective pore-space partitioning strategies),^43,44^ the diffusion rate of reactants/products through MOF channels could be controlled to accelerate catalytic cycles and potentially separate product mixtures.^45^ At the same time, using mixed-valence metal nodes and/or domains of conjugated linkers can enhance electronic conductivity, addressing challenges in applying MOFs in the area of electrocatalysis. For example, controlled chemical heterogeneity in separation membranes could create preferential adsorption gradients and specific free-volume distributions, providing a mechanism to overcome the classical trade-off between permeability and selectivity in conventional membrane design.^46^

Insights from drug delivery research further underscore the importance of framework heterogeneity, particularly given the potential to achieve precise temporal control over a material’s performance. Many of the challenges associated with MOF-based drug delivery platforms, such as rapid, uncontrolled drug release, arise either from uniform activity/interaction between guest drug molecules and the MOF, or from unanticipated adsorption of guest molecules onto MOF surfaces. Introducing material domains with varying binding strength, degradation kinetics, or stimuli-responsive behavior offers a route to staged or gradient drug-release profiles.^47^ For example, pH-labile or redox-sensitive linkers could enable sequential release of a pharmaceutical under biologically relevant triggers, reducing its premature leakage while preserving therapeutic efficacy.^48^ At the same time, framework “heterogeneity” could also resolve ongoing challenges associated with MOF-based sensors, such as limited analyte selectivity and slow response times. For example, chemical heterogeneity caused by open metal sites, defect-rich materials, and mixed linker domains could create many chemically distinct binding pockets capable of distinguishing between structurally similar analytes.^49^

Some key opportunities for further research include introducing chemical heterogeneity in a predictable and/or controlled manner, as well as integrating chemically heterogeneous MOFs into larger device architectures. Chemically heterogeneous MOFs could be integrated into composite or hybrid systems, where molecular-level heterogeneity within the framework is coupled to mesoscale or macroscopic heterogeneity in polymers, soft matter matrices, or conductive supports. Such multiscale architectures may address challenges related to mechanical fragility, processability, and electrical conductivity, critical limitations that currently restrict practical deployment of some classes of MOFs.^50^ By intentionally designing chemically heterogeneous microenvironments that couple distinct processes in space and time, MOFs can evolve into more adaptive and biomimetic chemical systems. Realizing this vision will demand close integration of synthesis, characterization, and theory, particularly through the development of spatially resolved techniques capable of (i) mapping linker distributions, (ii) defect populations, and (iii) local chemical environments across multiple length scales.

## “Smart” Adaptive Frameworks

MOFs already represent an important and growing class of adaptive materials that can undergo distinct changes in their physical, electronic, and magnetic properties upon exposure to external stimuli, such as pressure, light, temperature, current, or pH.^51,52^ For example, flexible or “breathing” MOFs are a well-established class of materials that undergo expansion and contraction of their pore volumes upon adsorption/desorption of gas/vapor or under an applied pressure.^53,54^ Similarly, incorporation of stimuli-responsive linkers within MOF structures has afforded noninvasive control over framework conductivity, optical profile, energy/charge transfer pathways, catalytic activity, and metal binding behavior through, for instance, exposure to appropriate excitation wavelengths.^55–58^ In light of the existing progress in the area of adaptive MOFs, two of the key areas that will likely experience significant growth in the near future are light-based (photonic) data storage and logic-gated (opto)­electronics.^55,59^ Both of the mentioned areas will rely on MOFs which exhibit significant changes in a measurable property (an “output”) as a function of an external stimulus (an “input”, Figure 2). For example, proof-of-concept for MOF-based logic gates has already been demonstrated through incorporation of guest molecules exhibiting optical nonlinearity as well as photochromic guests within the pores of conductive frameworks.^60,61^

**Figure 2:**
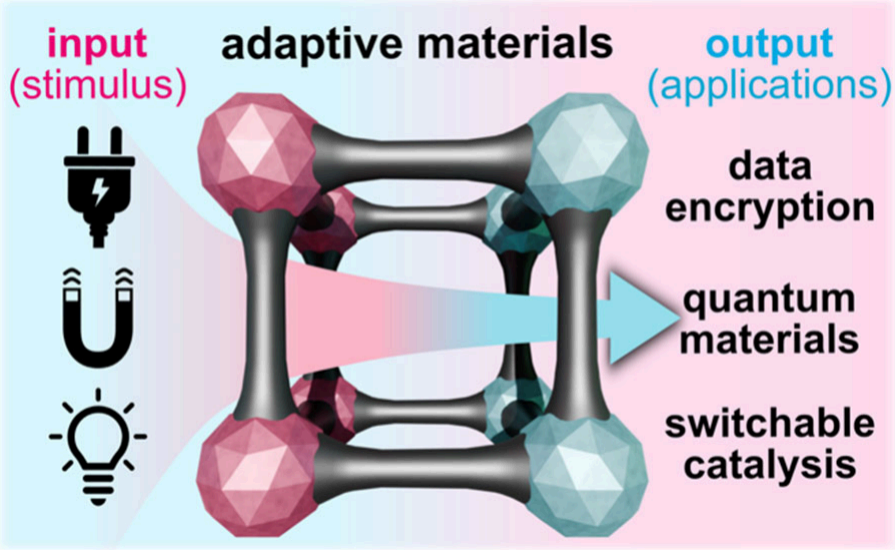
Schematic representation of an adaptive MOF which utilizes multiple possible external stimuli (e.g., current, magnetic field, or light) as an “input” to control material properties, leading to desirable applications as an “output”.

However, transitioning from proof-of-concept to practical adaptive MOF-based computing and data storage requires addressing several key challenges. For instance, many photoresponsive MOFs exhibit relatively low optical switching ratios (i.e., ≪100% conversion between two or more states), which limits their applicability in the existing technology sector where materials exhibiting clear binary (on/off) states are generally considered standard. At the same time, the selection of stimuli-responsive units (and corresponding external stimuli) must be compatible with device fabrication. For example, exposure of a flexible MOF to extreme pressures or vapors to induce structural changes may not be suitable for (opto)­electronics development, and therefore, future work will likely involve expanding the utilization of alternative stimuli, such as light, electric current, or magnetic field, to drive property changes of adaptive MOFs.^53,59,62^ In addition, integration of multiple stimuli-responsive units into a single material could be an avenue to achieve either cooperative or orthogonal behavior (e.g., stimuli responsive units responding to the same stimulus or two different stimuli).^53,63,64^ The ability to selectively activate two or more stimuli-responsive moieties using orthogonal stimuli, for instance, could allow for multivariable control over material properties in the future.^55,63^

An equally important direction to pursue is the integration of stimuli-responsive MOFs into larger device architectures while preserving the ability to achieve significant changes in material properties (e.g., optical profile, porosity, conductivity, etc.) in response to a stimulus, which will require collaborative effort between the chemistry and engineering communities. For example, many recent reports have suggested that photochromic frameworks may be suitable materials for photonics and optical data storage,^55^ but the mentioned applications strongly rely on advanced interfacial engineering, which could be a significant bottleneck in device fabrication. More importantly, the device structure must be optically transparent to the excitation wavelengths used to induce stimuli-responsive behavior. Advances in this area will not only demand design and fabrication of new MOF-based devices but also fundamental studies of the processes occurring at MOF-device interfaces (e.g., charge transfer, potential surface reactions, adhesion, etc.).

In the area of MOF-based electronics, logic gates attract significant attention and are likely to continue evolving in the coming years.^65,66^ In general, MOF-based logic gates operate by using two or more stimuli (inputs) to induce a measurable property change (output; Figure 2). Depending on the specific framework design, each stimulus could induce a given property change (known as an “OR” logic gate), or two simultaneous stimuli could be required to induce one output (known as an “AND” logic gate).^55,59^ In either case, it is critical for the measured property change (e.g., absorbance profile, pore size, conductivity, etc.) to be selective to a specific external stimulus. For instance, many flexible MOFs (as described above) undergo similar structural transitions under applied pressure or upon exposure to gases or vapors. As a result, pressure and vapor would not be suitable stimuli for construction of some types of logic gates since both “inputs” would cause the same “output”. Therefore, the development of adaptive frameworks that exhibit stimulus-specific transitions between two or more states (i.e., a single stimulus selectively induces a specific change in material properties) is the next step in applying MOFs in the design of logic-gated electronics.^65^

From a broader perspective, merging the MOF field with electronics design and computer science will require development of a common language and symbolism that unites many distinct disciplines.^55,59,65^ For example, existing terminology and symbolism within the computer science community may not be directly applicable or intuitive for chemists in the MOF field. Likewise, jargon commonly used to describe adaptive MOFs (e.g., “large-to-narrow” pore transitions) may not be suitable language for defining a logic gate in a manner meaningful to other fields, such as computer science. The described need for a more common language highlights that the future of MOFs in (opto)­electronics and logic-gated computing may offer many new research avenues, but, perhaps more importantly, it also illustrates an ongoing shift toward a more interdisciplinary approach to MOF research. Future advances in the MOF field will require inclusion of more diverse experts beyond the disciplines traditionally central to materials development, such as chemistry and materials science, and will most likely be driven by innovations from computer science, information technology, engineering, and other fields.

Future advances in the MOF field will require inclusion of more diverse experts beyond the disciplines traditionally central to materials development, such as chemistry and materials science, and will most likely be driven by innovations from computer science, information technology, engineering, and other fields.

## Emerging Applications

Since their inception, MOFs have been consistently applied to address environmental issues related to sensing, capture, and remediation of chemical contaminants, especially in the areas of direct air capture of CO_2_, hazardous gas storage, and detection of heavy metal pollutants in water.^5,12,14,15,67,68^ In the near future, however, one could anticipate that the role of MOFs and other MOF-based materials in the environmental remediation sector will be expanded to address urgent challenges related to nuclear waste (re)­processing and extraction/recycling of critical minerals (Figure 3). Both of the mentioned areas are growing rapidly due to increasing global demand for more energy- and resource-efficient industrial practices, as well as the allocation of federal funding for nuclear energy and critical mineral extraction research.^69–71^ While a few fundamental studies of MOFs constructed from building units containing radioactive species already exist, primarily in the form of Th- and U-based MOFs,^69^ the current literature related to frameworks that are either constructed from or can bind transuranic species is extremely limited.^72^ The significant knowledge gap in the area of actinide-based (including transuranics) MOFs can at least partially be attributed to the strict safety protocols and regulations regarding radioactive material handling. As a result, addressing the described knowledge gap will require long-term collaboration among teams of specialists, including trained nuclear engineers, radiochemists, and synthetic inorganic and materials scientists. In this direction, experimental evaluation of how factors such as pH, ionic strength, and temperature affect radionuclide binding within MOFs will be vital, given that materials used for nuclear waste processing are exposed to extreme conditions that are not readily simulated in a typical laboratory environment.

**Figure 3:**
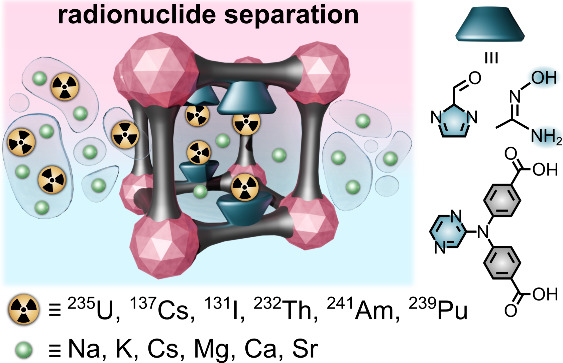
(Left) Schematic representation of a MOF designed to selectively bind radionuclides in the presence of competing ions (green spheres). (Right) Examples of several binding motifs that could be used for radionuclide sequestration. The possible radionuclide binding sites are highlighted in blue.

Outside of strengthening fundamental understanding of interactions between porous frameworks and transuranic species, an alternative direction to pursue is the development of efficient strategies for the release of isolated radionuclides from a MOF structure without material degradation (Figure 3). Many previous studies have focused on the capture of radioactive species from air (e.g., ^131^I), waste streams (e.g., ^137^Cs), or seawater (e.g., ^235^U), but very few have attempted their selective and controlled release from the framework for future recycling.^69,73,74^ While optimizing the storage capacity and selectivity of a MOF designed for nuclear waste remediation applications is certainly important, the future of MOFs in the nuclear energy sector will rely on robust materials that are designed to capture, separate, and release radioactive species on demand over many cycles without structural degradation.

In addition to designing MOFs for nuclear waste remediation, the growing challenge of recycling critical minerals used, for instance, in electronics or battery production (i.e., Li, Co, or Ni) will likely also impact the MOF field.^70,72,75^ Proof-of-concept for MOF-based separations of rare-earth elements, as well as elements such as Li and Sb, already exist but are challenged by the fact that the concentrations of critical minerals in natural sources and waste streams are often significantly lower than those of competing ions.^70,75^ Further, competing ions often possess the same oxidation states and similar ionic radii as the target critical minerals, and, therefore, design of organic linkers with extremely high and selective binding affinities for specific elements will continue to be relevant to the MOF field. These endeavors will likely be supported by advanced X-ray diffraction techniques, enabling the crystallographic resolution of specific binding interactions between critical minerals and frameworks, and their subsequent evaluation using computational modeling.

To realistically deploy MOF-based materials in either the nuclear waste or critical mineral recycling fields, significant effort will be required to integrate MOFs into highly processable matrices, such as polymers or mixed-matrix membranes, to form composite/hybrid structures, and to reliably produce high-quality thin films. Importantly, such studies must account for the fact that framework morphology (e.g., powders, pellets, single crystals, or thin films) is likely to impact material performance, highlighting yet another area that could be supported by more fundamental materials chemistry research in the future.

While novel MOF-based materials will definitely be critical platforms for addressing the ongoing energy crises, it is also important to recognize that MOFs may play an important role in unlocking next-generation technologies that rely on quantum materials (Figure 2). In particular, magnetic MOFs are expected to be key platforms for exploring concepts such as quantum spin liquids, as well as for materials exhibiting superconductivity and exotic topological states.^76,77^ For example, magnetic interactions between adjacent metal nodes could be tailored using organic linkers with varying lengths and shapes, as well as by incorporating stimuli-responsive linkers or organic molecules within the MOF structures that facilitate charge transfer/separation.^78^ As a result, the rational design of metal node connectivity and framework topology can result in preparation of materials exhibiting frustrated magnetism (i.e., possessing competing exchange interactions between adjacent spins that cannot be simultaneously satisfied).^76^ Fundamental studies of spin-frustrated frameworks could lead to formation of quantum spin liquids and/or materials exhibiting high-temperature superconductivity.^76,77^ However, existing studies of geometrically frustrated magnetism in MOFs are mainly limited to 2D frameworks, with a few notable exceptions.^77,79,80^ Thus, ongoing work will likely focus on developing design principles for 3D, spin-frustrated, MOF-based materials, as well as further detailed studies of nongeometrically imposed magnetic frustration (e.g., Kitaev-type materials exhibiting bond-directional Ising exchange interactions).^77,81^

In addition to frustrated magnetism in MOFs, an equally interesting avenue could be the design of frameworks that support quantum spin coherence even at room temperature, for instance, by strategically incorporating chromophores that promote singlet fission.^82^ Such platforms could behave as qubits (the basic unit of information in quantum information science) and may be the key to unlocking the next generation of materials for quantum computing.^82,83^ However, progress in this area is currently limited by a lack of fundamental mechanistic studies (e.g., performed at both low and room temperatures) as well as challenges associated with large-scale single-crystal growth or the preparation of defect-free samples of magnetic MOFs.^78^ An alternative direction to pursue is the synthesis of 2D (atomically thin) samples grafted to substrates possessing variable electronic properties which could be used to tailor the performance of the hybrid material, as suggested by Armitage and co-workers.^83^ Finally, while relatively underexplored, a few previous reports of quasicrystalline and quasiperiodic MOFs have shown great promise as quantum materials and represent an exciting direction to pursue.^84–86^ Overall, fundamental studies of quasicrystalline frameworks, magnetic MOFs, and quantum materials represent two highly active and promising areas of research with vast potential for collaboration among chemists, materials scientists, and physicists.

## MOFs at the Global Scale: Commercialization and Device Deployment

As of the end of 2025, more than 45 startup companies (e.g., Attoco, NuMat, Transaera, and novoMOF) have already brought MOFs to the industrial scale and deployed MOF-based devices for indoor climate control and dehumidification, decontamination of chemical warfare agents, water harvesting in arid regions, and direct air capture of CO_2_, among other applications.^16,37,87–91^ Thus, it is clear that MOF synthesis can be compatible with multikilogram-scale production (or even kiloton-scale in select cases). For instance, ZIF-8, UiO-66, MIL-53­(Al), and CALF-20 (ZIF = zeolitic imidazolate framework; UiO = University of Oslo; MIL = Materials of Institute Lavoisier; and CALF = Calgary Framework) can all be reliably synthesized at the industrial scale.^92^ However, several different research directions could bring a wider array of MOFs to the commercial scale, including (i) large-scale syntheses that do not sacrifice MOF purity, crystallinity, and porosity, (ii) development of shaping methods to convert bulk powders or crystals into usable forms (i.e., macroscopic pellets, beads, films, or foams),^93^ and (iii) use of solvent-free procedures or environmentally benign solvent systems.^92,94^ At the same time, compatibility of MOF-based materials within existing industrial workflows could be greatly enhanced by fundamental studies of rheology as well as design of thermally processable/glassy frameworks.^92,93^ Additionally, methods for preparation of >1-m MOF solids (e.g., films, composites, or foams) would enable manufacturing of larger-scale MOF-based devices, but are currently rarely explored in scientific literature (Figure 4). One of the important considerations at the heart of all MOF commercialization and device integration, however, is the long-term stability of high-performance frameworks with various morphologies, such as foams, beads, or pellets.^95,96^ For instance, many MOFs already possess exceptional thermochemical stability under harsh conditions, but further *in situ* and *in-operando* studies of their stability after shaping, integration within devices, and upon repeated device operation are necessary.^97^

**Figure 4:**
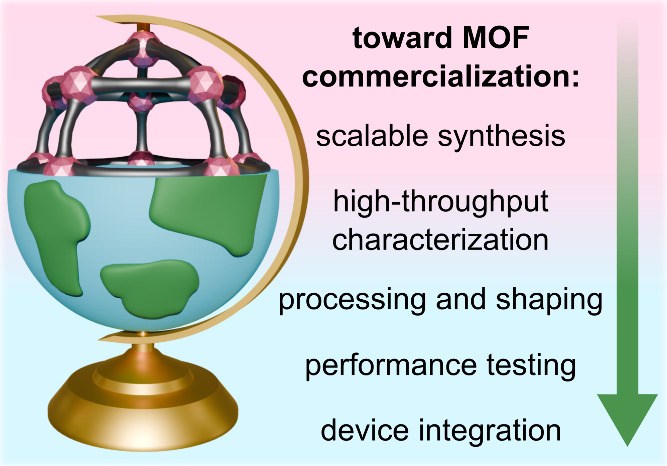
Summary of key steps for advancing global MOF commercialization and deployment of MOF-based materials and devices.

Beyond the development of new chemical processes to bring a broader library of MOFs to commercial scale, an alternative approach that will continue to evolve is the development of high-throughput and automated systems for MOF preparation (Figure 4). For instance, there are limited existing examples of high-throughput MOF synthesis enabled by automated liquid handling. However, the majority of current MOF synthetic protocols require transferring small amounts of solids with high accuracy. Therefore, a fully automated and generalizable workflow for MOF synthesis is not currently broadly accessible.^98^ At the same time, characterization of the resulting solid MOF products after reaction completion is not trivial and may be difficult to automate, especially considering that many preliminary synthetic attempts may result in polymorphic or phase impure samples.^98^ In such cases, expert analysis rather than automated interpretation of, for instance, powder X-ray diffraction data, may be necessary.^98^ Therefore, there is currently a significant engineering gap related to material development that restricts automated MOF preparation in an industrial setting that will be addressed through collaborative work in the near future.^6,87,99^ However, it is important to emphasize that significant progress has already been made in the direction of automated and/or high-throughput MOF preparation and characterization, supporting the idea that solutions to the mentioned challenges are not only possible but are actively being pursued.^100–104^

Over the past several years, there has been an increasing effort to apply computational analysis, artificial intelligence (AI), and machine learning (ML) methods to accelerate the discovery of novel functional materials and potentially to automate MOF preparation workflows. The use of AI to guide experiments, especially for synthesizing new MOF structures, has already shifted how chemists approach material development. For instance, the ability to prescreen combinations of metal nodes and organic linkers to target a particular structure or property can drastically reduce the number of trial-and-error synthetic conditions needed to prepare novel MOFs.^98,105–107^ As a result, AI-guided MOF synthesis minimizes both time and resources usually spent during the research and development process. In many cases, advanced MOF synthesis requires the use of highly complex, functionalized organic linkers prepared through labor-demanding, multistep synthetic protocols and, in some cases, relatively precious metal salts, which would be advantageous to conserve during synthetic trials. Following framework synthesis, the performance metrics extracted from experimental data (e.g., surface areas, catalytic activity, gas sorption, etc.) can be used as a starting point for the next iteration of predicted framework structures. As a result, AI (and ML) models can accelerate the “structure-property-performance” loop for materials discovery.^98,105^

Despite the advantages of AI-guided experimental research, several aspects must be addressed to fully leverage current and upcoming technologies. In particular, a universal commitment to complete and accurate data reporting for both successful and failed synthetic attempts will be necessary. The described level of scientific transparency is not trivial, given that the majority of published data sets, understandably, represent only successful reaction conditions and physical measurements; experimental methods that did not yield the desired outcome are typically excluded from publication.^106,107^ However, unsuccessful or “failed” experiments are extremely valuable for guiding global research efforts conducted in both laboratory settings and AI model training. Additionally, subtle changes in experimental conditions which may go unnoticed, such as relative humidity, solvent grade, and ambient temperature, can significantly impact the formation of crystalline structures and/or product morphology.^98,107^ In the case of using AI models to select suitable synthetic conditions for a MOF targeted toward heterogeneous catalysis, for instance, the experimental outcomes could be drastically altered by particle size.^60,62^ Therefore, consistent, complete, and accurate data reporting in a standardized format is necessary for facile advancements in AI-guided MOF design.^98,106^

## Conclusion

To summarize, this Outlook highlights only a few of the possible concepts that are actively transforming all aspects of the MOF landscape, ranging from material design and synthesis to advanced applications and commercialization. For example, development of synthetic strategies for preparation of frameworks with controlled chemical heterogeneity is an exciting direction that could advance heterogeneous tandem catalysis and gradient drug delivery systems. Moreover, continued research efforts to apply MOFs as potential solutions for urgent global energy and environmental challenges will likely expand toward studies of actinide-based and, in particular, transuranic frameworks. Preliminary studies of actinide-containing MOFs have already demonstrated their potential as materials for selective radionuclide capture and separation, aspects that will likely define the future of the nuclear sector. Alternatively, there are urgent areas for implementing MOFs in extraction and recycling technologies focused on critical minerals used in electronics. At the same time, the MOF field is poised to more directly impact the technology sector through design of adaptive “smart” and/or quantum materials for next-generation data storage, encryption, and computing. Finally, we also highlight the importance of addressing some of the “missing steps” in MOF commercialization, such as development of high-throughput or AI-guided synthesis and characterization methods.

While the principles of reticular chemistry may have been born in the materials and supramolecular chemistry communities, the future of MOFs beyond the Nobel Prize lies in creative collaboration among physicists, engineers, computer scientists, biologists, mathematicians, and chemists alike.

In addition to the emerging research directions described above, the broader MOF field will continue to expand, incorporating contributions from a wider variety of disciplines. While the principles of reticular chemistry may have been born in the materials and supramolecular chemistry communities, the future of MOFs beyond the Nobel Prize lies in creative collaboration among physicists, engineers, computer scientists, biologists, mathematicians, and chemists alike. Merging the key strengths of the fields mentioned will undoubtedly usher in a new era of framework science that relies not only on synthetic chemistry research but also on global innovations across all scientific disciplines.

## Abbreviations

MOF: metal–organic framework
UiO: University of Oslo
MIL: Materials of Institute Lavoisier
CALF: Calgary Framework
ZIF: zeolitic imidazolate framework
